# Eye movement impairments in Parkinson's disease: possible role of extradopaminergic mechanisms

**DOI:** 10.1186/1471-2377-12-5

**Published:** 2012-02-29

**Authors:** Elmar H Pinkhardt, Reinhart Jürgens, Dorothée Lulé, Johanna Heimrath, Albert C Ludolph, Wolfgang Becker, Jan Kassubek

**Affiliations:** 1 Department of Neurology, University of Ulm, Oberer Eselsberg 45, 89081 Ulm, Germany; 2 Department of Neurology, Section Neurophysiology, University of Ulm, Ulm, Germany

**Keywords:** Deep brain stimulation, Parkinson's Disease, Oculomotor function, Neurophysiology, Eye movement, Neurodegeneration

## Abstract

**Background:**

The basal ganglia (BG) are thought to play an important role in the control of eye movements. Accordingly, the broad variety of subtle oculomotor alterations that has been described in Parkinson's disease (PD) are generally attributed to the dysfunction of the BG dopaminergic system. However, the present study suggest that dopamine substitution is much less effective in improving oculomotor performance than it is in restoring skeletomotor abilities.

**Methods:**

We investigated reactive, visually guided saccades (RS), smooth pursuit eye movements (SPEM), and rapidly left-right alternating voluntary gaze shifts (AVGS) by video-oculography in 34 PD patients receiving oral dopaminergic medication (PD-DA), 14 patients with deep brain stimulation of the nucleus subthalamicus (DBS-STN), and 23 control subjects (CTL);In addition, we performed a thorough review of recent literature according therapeuthic effects on oculomotor performance in PD by switching deep brain stimulation off and on in the PD-DBS patients, we achieved swift changes between their therapeutic states without the delays of dopamine withdrawal. In addition, participants underwent neuropsychological testing.

**Results:**

Patients exhibited the well known deficits such as increased saccade latency, reduced SPEM gain, and reduced frequency and amplitude of AVGS. Across patients none of the investigated oculomotor parameters correlated with UPDRS III whereas there was a negative correlation between SPEM gain and susceptibility to interference (Stroop score). Of the observed deficiencies, DBS-STN slightly improved AVGS frequency but neither AVGS amplitude nor SPEM or RS performance.

**Conclusions:**

We conclude that the impairment of SPEM in PD results from a cortical, conceivably non-dopaminergic dysfunction, whereas patients' difficulty to rapidly execute AVGS might be related to their BG dysfunction.

## Background

A broad variety of oculomotor alterations have been described in Parkinson's disease (PD) such as an increased latency of visually guided reactive saccades, reduced saccadic gain, impaired smooth pursuit and difficulties to inhibit unwarranted reactions [1]. Existing literature mainly focuses on different aspects of saccadic dysfunction and basal ganglia pathology without arriving at a generally accepted view. The substantia nigra and related brainstem areas are suggested to be crucially involved in various types of saccadic eye movements by mediating a dopamine-related descending input from frontal cortical areas to the superior colliculus [2], but are also thought to play a role in inhibiting inappropriate saccades [3]. To better understand the emergence of oculomotor alterations in PD, the effects of therapy on eye movements (dopaminergic treatment or deep brain stimulation of the subthalamic nucleus, STN-DBS) have been repeatedly examined. Some studies show no effect of dopaminergic medication [4], whereas other studies do show effects on latency, gain or amplitude of saccades (e.g. [5]). In general, dopaminergic treatment is supposed to be of little effect on ocular motor deficits in PD [6], in contrast to its benefit for limb motor function. Regarding the effects of STN-DBS on saccadic function, there exist only a few studies, so far. Most of these report a reduction of the latency of visually elicited reactive saccades (RS) [2] as well as an increase in gain [7,8], whereas another study found no effect on latency and gain of RS [9] but an improvement of the amplitude of memory guided saccades [8]. Finally, a single case report suggests that saccadic intrusions in patients can be reduced by STN-DBS [10]. Since STN-DBS is generally accepted to be highly effective in reducing the levodopa sensitive parkinsonian motor symptoms [11], these observations suggest that oculomotor deficits in PD might to a considerable part be caused by a dysfunction of non-dopaminergic systems.

There is ample evidence that PD is a multisystem disorder with involvement of several brain regions and neurotransmitter pathways other than dopamine [12]. Hence the cause of numerous clinicopathological correlates of PD-related deficits such as cognitive dysfunction, dementia, depression, and behavioural or emotional dysfunction have to be searched outside the nigrostriatal system. Recently, these neuropsychological and psychiatric changes in PD have received considerable attention. They are supposed to arise either as a part of the disease itself but also as a collateral result of STN-DBS [13]. If non-dopaminergic deficits cause indeed some of the eye movement alterations in PD, it might be possible to demonstrate a correlation between such non-motor symptoms and oculomotor impairment in PD.

Since a number of methodological concerns complicate the interpretation and comparison of studies that examine the impact of dopaminergic therapy (different testing procedures, failure to counterbalance 'on' and 'off' order of testing, different dopaminergic medication and withdrawal regimes [14]), we chose to study PD patients with STN-DBS in the "on" and "off" condition and medication "on", but also PD patients with exclusively oral dopaminergic medication. Patients' eye movement were recorded by video-oculography (VOG) and analysed in relation to their demographic, clinical and neuropsychological data.

## Methods

### Patients and controls

Fourteen patients with the diagnosis of PD receiving STN-DBS (PD-DBS), 34 PD patients with exclusively oral dopaminergic medication (PD-DA), and 23 age matched controls (CTL) free of any neurological disease were examined by VOG. The relevant demographic details are listed in Table 1. All subjects gave their informed consent, and the study was approved by the local ethics committee.

**Table 1:** Demographic data, medication (levodopa equivalent) and clinical scores.

	CTL	PD-DA	PD-DBS	
			**on**	**off**

*Number of subjects* total (male, female)	23 (18m, 5f)	34 (21m, 13f)	14 ( 9m, 5f)	

*Age (yrs)* mean (± SD), range	61.7 (± 7.1), 49-76	63.0 (± 11.7), 36-81	64.2 (± 7.1), 50-74	

*Disease duration (yrs)* mean (± SD), range		6.9 (± 4.4), 1-16	10.5 (± 4.0), 4-19	

*Levodopa equivalent medication (mg/day)* mean (± SD), range		917 (± 578), 200-2650	509 (± 348), 0-1250	

*UPDRS motor scale* mean (± SD), range		20.3 (± 13.0), 2-56	29, 0 (± 16.3), 5-62	38.8 (± 14.3), 16-64

Equivalent doses are given according to Tomlinson et al., 2010 [15]

The diagnosis of PD was made according to the UK Parkinson's Disease Society (UKPDS) Brain Bank criteria [16]. All patients were assessed and diagnosed by a board-certified neurologist specialized in movement disorders. The Unified Parkinson's Disease Rating Scale (UPDRS) Part III (motor assessment) was performed in all PD-DBS and 23 PD-DA patients. The PD-DBS patients were examined with medication "on" in the conditions stimulation "on" (PD-DBS-on) and stimulation "off" (PD-DBS-off).

### Neuropsychological assessment

In all PD-DBS patients and in 10 of the PD-DA patients, neuropsychological tests were conducted which included a recognition vocabulary test of general intelligence ("Wortschatztest", WST) and assessments of dementia (Parkinson Neuropsychometric Dementia Assessment, PANDA), depression (German version of the Geriatric depression scale, GDS) and susceptibility to interference (Stroop colour-word-test/"Farbworttest", FWT). As a score of susceptibility to interferences pointing to frontal failure, FWT3-2 was obtained from the difference between the times required for subtests 3 and 2 of FWT (in s).. Learning in decision making was evaluated with a PC version of the IOWA gambling task (IGT) In DBS, all tests except IGT were only performed during "on"; the IGT was also performed 30-45 min after suspending stimulation.

### Eye movement recording and analysis

The investigation took place in an optically and acoustically shielded room. Subjects were seated, with their heads stabilised by a chin rest, in a comfortable chair at the centre of a hemicylindrical screen (radius, 160 cm). The screen carried a horizontal array of red light emitting diodes (LEDs; spaced 5°, up to 20° right and left; invisible when not lit). Visually guided reactive saccades (RS) were elicited by randomly lighting one of these LEDs as a target in such way that target steps of 5, 10, 20, and 40° resulted. Self-paced rapidly left-right alternating voluntary gaze shifts (AVGS) were obtained by asking subjects to saccade back and forth as frequently as possible for 30 s between two permanently lit red LEDs spaced 20° horizontally. Smooth pursuit eye movements (SPEM) in horizontal and vertical direction were elicited by sinusoidal movements of a red laser spot across the screen (horizontal amplitude ± 20°, vertical ± 15°, frequencies 0.125 and 0.375 Hz). The movements of both eyes were recorded with a VOG system (EyeLink I^®^, SR Research Ltd., Osgoode, ON, Canada) at a sampling rate of 250 Hz and an effective angular resolution of about 0.05°. The restriction to horizontal saccades was necessary to limit the period of time the severely handicapped DBS patients would spend in the "off" condition. According to [17], after suspending stimulation the beneficial effect of DBS-STN on akinetic, rigid and tremor symptoms decreases to reach a plateau after 30 min without any further worsening within two more hours. Therefore, recording in the "off"-state originally was intended to start 45 min after stopping stimulation; however, depending on the severity of the patients' symptoms in the "off"-state, recording had to be started between 30 and 45 minutes after stopping stimulation, as otherwise some patients would have not been able to complete the examination. The order of recording during "off" and "on" was randomised across patients; "on"-recordings following an "off"-state were commenced 45 min after resumption of stimulation.

For analysis, the following parameters were extracted from the left and the right eye recordings, respectively: (1) SPEM gain (fundamental component of SPEM velocity/target velocity), (2) peak velocity of RS of 20° amplitude (by interpolation along the "main sequence"), (3) latency and (4) gain of RS aimed at targets of 20° retinal eccentricity (amplitude of primary saccade/20°), (5) number N30 and (6) gain G(N30) of AVGS executed within 30 s where G(N30) was calculated from the largest saccade of each gaze shift.

### Statistical analysis

None of the groups exhibited systematic differences between the right and the left eye. Therefore, analyses are based on averages of the monocular parameters. To detect an effect of group, all oculomotor parameters were subjected to a Kruskal-Wallis analysis of variance (ANOVA) with those of DBS being taken from the "on" state (PD-DBS-on) as the best therapy equivalent of PD-DA. Suspected differences between these groups were analysed with the Mann-Whitney *U *test with probabilities being Bonferroni corrected according to the number of comparisons. PD-DBS-off was compared to PD-DBS-on by a matched pairs Wilcoxon tests. To check for neuropsychological differences between PD-DA and DBS-on, the Mann-Whitney *U *test was used. For possible correlations between oculomotor, clinical, and neuropsychological data, Spearman's rank correlation coefficient was used. A threshold of *p *< 0.05 was adopted for all statistical inferences.

## Results

### Clinical data

Most DBS patients continued to receive a regular adjuvant dopaminergic medication after surgery. However, its dose could be considerably reduced as compared to the presurgical level (average levodopa equivalent, 509 mg/day) At the time of eye movement recording, their average UPDRS part III score improved from 38.8 during "off" to 29 during "on" (*p *< 0.001) And so did the subscore for rapid alternating hand movements (2.7 versus 2.1, *p *< 0.018).. PD-DA patients had received L-DOPA equivalents of 917 mg/day [15] and reached an average UPDRS score of 20.3 (2.0 for the hand movement subscore). For details of medication and UPDRS see Table 1.

### Neuropsychological assessment

There were no significant differences in the applied tests between PD-DBS-on and PD-DA except for a lower GDS score in the PD-DBS-on patients (PD-DA: mean 4.5 ± 2.7; PD-DBS-on: 3.3 ± 2.8; *p *= 0.021). Gambling score IGT did not differ between "on" (mean 4.2 ± SD 21) and "off" (3.4 ± SD 30) conditions in PD-DBS patients.

### Eye movement data

**Figure 1:**
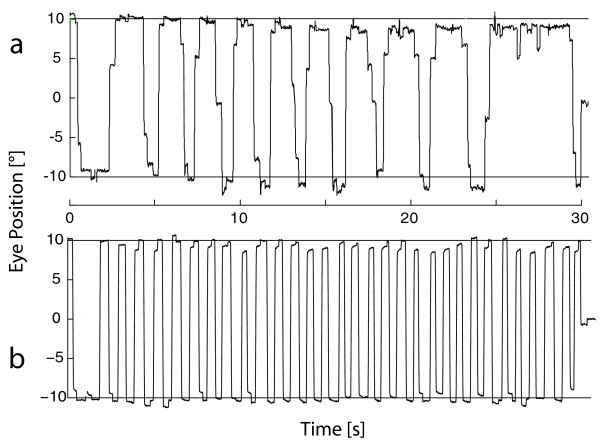
**Sample records of self-paced gaze shifts alternating as rapidly as possible between two permanently lit targets subtending 20° (AVGS)**. a, DBS-off patient; b, control.

ANOVA indicated a significant effect of group (CTL, PD-DA, PD-DBS-on) on most of the investigated eye movement parameters (Table 2); exceptions were downward pursuit gain, saccade gain and saccade peak velocity. These effects were due to significant differences between both PD-DA and PD-DBS-on on the one hand and CTL on the other hand, whereas PD-DA and PD-DBS-on did not differ between each other. Specifically, SPEM gain and the number and gain of voluntary saccades produced within 30 s (N30) was markedly reduced in patients (Figure 1), whereas saccade latency (only PD-DA) increased. No parameter changed significantly when stimulation was switched off in DBS patients except for N30 which exhibited a just significant decrease (*p *= 0.046).

**Table 2:** Oculomotor results.

	Smooth pursuit gain						Saccades				
	**0.125 Hz**			**0.375 Hz**			**Visually guided reactive**			**Alternating voluntary**	

	**horiz**.	**up**	**down**	**horiz**.	**up**	**down**	**L (ms)**	**G20**	**Vpk (°/s)**	**N30**	**G(N30)**

CTL	0.98	0.94	0.89	0.92	0.89	0.57	245	0.91	419	62	0.96
	
	0.91-1.02	0.75-0.98	0.61-1.02	0.55-0.98	0.46-1.00	0.26-0.77	205-323	0.87-0.98	312-473	50-79	0.94-0.99

PD-DA	0.91*	0.87	0.87	0.77*	0.54*	0.48	296*	0.88	424	48*	0.89*
	
	0.60-.99	0.57-1.01	0.55-0.99	0.19-0.94	0.16-0.91	0.24-0.82	214-502	0.76-0.97	330-502	21-68	0.75-0.98

PD-DBS-on	0.89*	0.71*	0.77	0.74*	0.45*	0.42	268	0.88	390	28*	0.87*
	
	0.47-.98	0.51-.95	0.53-0.97	0.31-0.95	0.11-0.83	0.22-0.79	223-424	0.78-0.93	288-501	23-70	0.81-0.93

PD-DBS-off	0.83	0.66	0.71	0.66	0.38	0.31	288	0.88	389	23^+^	0.82*
	
	0.58-1.00	0.39-0.97	0.52-1.03	0.24-0.97	0.17-0.84	0.14-0.86	226-446	0.68-1.01	284-494	13-69	0.74-1.03

Median values with 5%-95%-ranges. L, latency; G20, gain of reactive saccades (RS) aimed at targets of 20° eccentricity; Vpk, peak velocity of RS with 20° amplitude (obtained by interpolation along the main sequence); N30 and G(N30), number and gain rapidly alternating back and forth gaze shifts during 30s-epoch (AVGS). *, significant differences with respect to CTL (Kruskal-Wallis-ANOVA of groups CTL, PD-DA and PD-DBS-on followed by Mann-Whitney group comparisons, Bonferroni corrected for multiple testing; all cases *p *< 0.005) except saccade latency of PD-DA vs. CTL (*p *< 0.01); note that there were no significant differences between PD-DA and PD-DBS-on. ^+^, significant difference (*p *< 0.05) between PD-DBS-on and PD-DBS-off (Wilcoxon matched pairs); note that PD-DBS-off was not compared to CTL or PD-DA to avoid repeated measurements

### Correlation analyses

**Figure 2:**
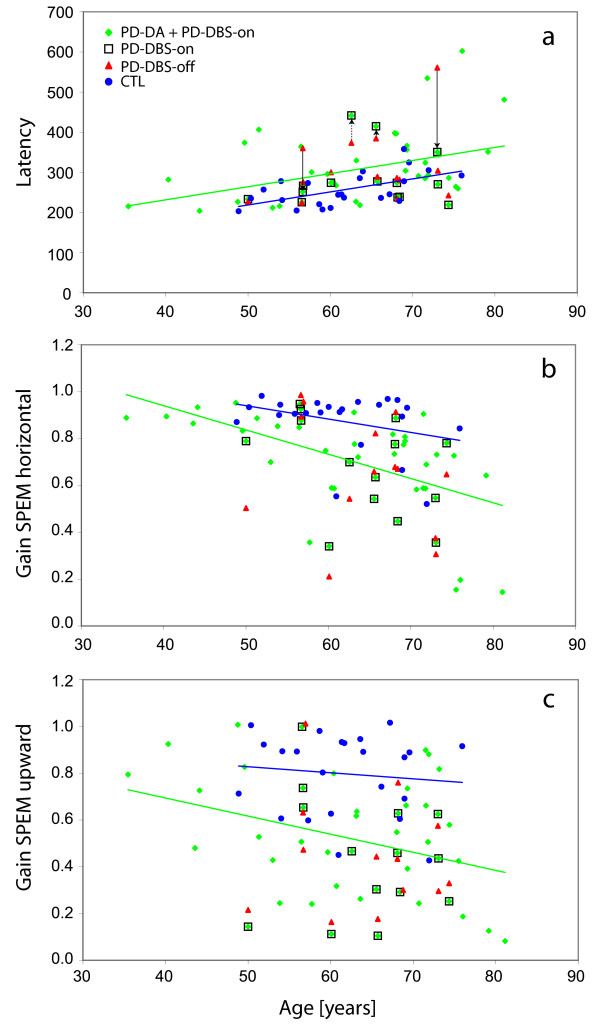
**Oculomotor performance as a function of age**. Blue filled circles, control subjects (CTL); green diamonds, patients with only dopamine therapy (PD-DA) or with additional STN-DBS (PD-DBS-on, green diamonds framed by black squares); red triangles, DBS patients during stimulation off (PD-DBS-off). a, latency of reactive saccades; arrows link "on"- and "off"-data of those 4 Patients who exhibited latencies outside the CTL-range during "off" (range accidentally delimited by latency-on-age regression of PD-DA + DBS-on, green line); solid black arrows mark near significant changes (*p *< 0.059); dotted arrows, non-significant changes. b, c, gain of horizontal (b) and upward (c) smooth pursuit of a sinusoidally moving target (0.375 Hz, horizontal ± 20°, vertical ± 15°).

To detect possible correlations between patients' oculomotor results, neuropsychological performance, demographic data and clinical scores, we pooled the data of groups PD-DA and PD-DBS-on as their oculomotor and neuropsychological variables were statistically indistinguishable (except for GDS). UPDRS part III correlated with none of the parameters and scores considered; also a comparison of the subscore for rapid alternating hand movements with the frequency (N30) and gain (G(N30)) of rapidly alternating gaze shifts (AVGS) revealed no correlation. Likewisedisease duration (except for a significant correlation with FWT3-2, cf. below) was uncorrelated to any of the parameters considered.. Also, no correlation existed in the DBS subgroup between the changes of UPDRS III and of its alternating hand movements subscore resulting from STN-DBS and the concomitant changes of eye movement parameters. FWT3-2 correlated negatively with low frequency (0.125 Hz) horizontal (r = -0.46, *p *= 0.025) and downward (r = -0.46, *p *= 0.028) SPEM gain and positively with disease duration (r = 0.51, *p *= 0.014), while PANDA correlated only with 0.125 Hz downward SPEM gain (r = 0.48, p = 0.017). Otherwise, age was an important factor in both PD and CTL; high frequency (0.375 Hz) SPEM gain decreased with age in PD (r < -0.34, *p *< 0.02 for horizontal and downward, trend for upward; Figure 2b, c) as did N30 (r = -0.40 *p *= 0.011), whereas RS latency increased with age (r = 0.36, *p *= 0.012; Figure 2a). Qualitatively similar trends held for the CTL group (significant for downward SPEM, r = -0.42 *p *= 0.046) and latency r = 0.57, *p *= 0.002). In the case of saccade latency, the regression lines of latency on age had virtually identical slopes in PD and CTL, with the former being shifted upward by about 60 ms (Figure 1a).

## Discussion

In this study we investigated whether an accurate assessment of oculomotor changes in PD patients - subdivided in a group with oral dopaminergic medication and a group of STN-DBS patients in the DBS "on" and "off" condition with additional oral dopaminergic medication - may allow a conclusion as to the pathomechanism of oculomotor dysfunction in PD. With regard to oculomotor function, we observed a reduced SPEM gain (typically caused by the release of predictive saccades, cf. Figure 1 in [18]), increased latency of RS (in PD-DA only) and a decreased number and gain of AVGS in all patient groups in comparison to controls. These results are in general agreement with the literature on oculomotor dysfunction in PD (i.e. [1]). However, none of the investigated oculomotor parameters exhibited significant differences between patients treated with dopamine only (group PD-DA) and those receiving STN-DBS (group PD-DBS-on). In DBS patients, the only effect of stimulation withdrawal was a just significant reduction of N30 whereas all other examined oculomotor parameters did not differ.

STN-DBS has been reported to significantly reduce the latency of visually triggered saccades [2,7,8], and to increase their gain [7,8]. Similar to the present study, Rivaud-Péchoux et al. observed an only non-significant shortening of latency by STN-DBS but a reduction of errors in an antisaccades task. A convincing mechanistic explanation for the observed improvements does not seem to exist, so far. Since STN neurones are thought to excite crossed nigro-collicular cells, it is difficult to understand how a supposedly elevated STN activity in PD could facilitate (instead of prevent) the release of inappropriate saccades by the colliculus observed in many studies [4,6] or how a reduction of STN overactivity by DBS could lead to the reduction of inappropriate saccades noted in other studies [10]. Sauleau et al. [7] invoke an altered control of the interaction between STN and substantia nigra pars reticulata (SNpr) in advanced PD which would be restored to normal via improved functioning of the parieto-collicular pathway. Temel et al. [19] suggest that the reduction of STN activity by DBS enhances the facilitation of the colliculus by interacting with the cortico-basal-collicular pathway. Yugeta et al. [8] invoke the more recent finding that PD pathology may be linked to the occurrence of abnormal oscillatory β-activity in STN. Similar to skeleto-motor performance [20], desynchronisation of this activity by STN-DBS would also be beneficial for oculomotor behaviour although its differential effect on reactive and unsolicited saccades remains to be explained.

Why do our measurements of saccadic latency not reproduce the beneficial effect of STN-DBS reported by others? With regard to latency and saccadic gain, the majority of our STN-DBS patients appear to have been too close to normal to exhibit any sizeable effect of STN-DBS. Moreover, the tendency for longer saccade latencies in PD as compared to CTL may in part have resulted from their dopaminergic medication that had been received two hours before eye movement recording [5].

We are unaware of studies that would have examined the effect of STN-DBS on the SPEM gain. The lack of a significant SPEM gain amelioration by STN-DBS in our patients suggests that the stimulation does not improve the inhibitory control of unwarranted saccades during pursuit (a main cause of SPEM gain reduction, [18,21-26]). Experimental evidence for an amelioration of inhibitory control from previous studies by STN-DBS is mixed, at best. STN-DBS was found ineffective at reducing antisaccade errors [8,9], but reportedly diminishes the frequency of premature saccades during a memory saccade task [8].

As a further paradigm which has not been studied during STN-DBS, we have examined the ability to rapidly saccade back and forth between two fixed targets. This paradigm was the only one to reveal a significant but small improvement under STN-DBS. This result is in line with the notion of the basal ganglia being particularly important for voluntary, endogenous saccades [3] in PD. In summary, our data suggest that STN-DBS can increase the frequency of voluntary saccades but does not improve SPEM gain in PD.

In contrast to the investigated eye movement parameters, the patients' skeletomotor system (UPDRS III scores) clearly improved with STN-DBS. The beneficial effect of STN-DBS on motor function is well established [11]. Nevertheless, if the observed eye movement alterations in PD resulted from a dysfunction of mainly dopamine-mediated mechanisms that can be relieved by STN-DBS, there should exist a correlation between STN-DBS-related improvement in UPDRS III and eye movement performance. We observed no such correlation, in particular when comparing the only eye movement parameter improving with DBS-STN (N30) to a skeletomotor analogue (UPDRS III subscore for rapid alternating hand movements). However, also authors who noted a SPEM improvement with dopamine agonists point out that this effect is much smaller than the concurrent UPDRS III improvement [22].

Also studies addressing the effect of dopamine on eye movements have produced ambiguous results (i.e. [4-6,23]). Some of these studies warrant methodological concerns such as different medication and withdrawal regimes (for a review, [14]). Moreover, the long half-value period of the ubiquitously applied dopamine agonists makes it difficult to examine patients in a true medication-off state since the medication-free time has to be limited for ethical reasons and because it is unclear whether there is a direct link between the plasma level of medication and possible influences on oculomotor function.

Compared with skeleto-motor function, there may be not an as clear a link between pathologic STN oscillations and eye movement alterations as these oscillations mainly affect dorsal STN and less so the ventral, eye movement related STN [25] Therefore, the effect of STN-DBS on eye movement alterations may be limited. Taken all together, the above considerations are in line with growing evidence that pathologies outside the dopaminergic nigrostriatal pathway may also be responsible for oculomotor deficits in PD (i.e. [26]). In view of the occurrence of frontal lobe pathology in PD [18], an impact of non-dopaminergic alterations in frontal cortical areas on eye movement control in PD is conceivable [27]. In this regard Rieger et al. [28] have found the frontal eye field itself to be hypoactive when performing horizontal voluntary saccades in an fMRI study. This study [28] as well as data showing a statistically significant correlation between the impairment of smooth pursuit and premature saccades in a delayed saccade task in PD [21] strongly support for the assumption of a top-down frontostriatal pathomechanism of saccadic dysfunction in PD..

## Conclusions

Available literature offers a mixed picture concerning the effects of therapy on eye movement control in PD, be it dopaminergic medication or high frequency deep brain stimulation. Some studies show no effect of dopaminergic medication [4], whereas others report effects on latency, gain or amplitude of saccades (i.e. [5]). In general, dopaminergic treatment has been found to be of little effect on the ocular motor deficits in PD [6]. STN-DBS has been reported to significantly reduce the latency of visually triggered saccades [2,7,8], and to increase their gain [7,8]. If eye movement alterations in PD resulted from a dysfunction of dopamine-mediated mechanisms exclusively, motor performance in general should correlate with eye movement performance both in terms of disease progression and therapeutical response. The lack of such correlation in our data supports the idea of a mainly non-dopaminergic top-down frontostriatal pathomechanism of oculomotor dysfunction in PD.

## Competing interests

The authors declare that they have no competing interests.

## Authors' contributions

EH P, RJ, DL, JH, ACL, WB and JK participated in the design and coordination of the study, helped in the acquisition of data, contributed substantially to the interpretation of data, drafted the manuscript and read and finally approved the final manuscript.

## Pre-publication history

The pre-publication history for this paper can be accessed here:

http://www.biomedcentral.com/1471-2377/12/5/prepub
